# Report of Mosquito Vectors of Arboviruses from a Federal Conservation Unit in the Atlantic Forest, Rio de Janeiro State, Brazil

**DOI:** 10.3390/life12101597

**Published:** 2022-10-13

**Authors:** Shayenne Olsson Freitas Silva, Cecilia Ferreira de Mello, Juan Augusto Rodrigues dos Campos, Paulo José Leite, Rebeca Sabino, Jeronimo Alencar

**Affiliations:** 1 Diptera Laboratory, Oswaldo Cruz Institute (FIOCRUZ), Av. Brazil 4365, Manguinhos, Rio de Janeiro 21040-360, Brazil; 2 Graduate Program in Tropical Medicine, Oswaldo Cruz Institute (FIOCRUZ), Av. Brazil 4365, Manguinhos, Rio de Janeiro 21040-360, Brazil

**Keywords:** Culicidae, *Haemagogus leucocelaenus*, eggs, climatic variables, breeding sites

## Abstract

Arbovirus infections, such as dengue, zika, chikungunya, and yellow fever, are a major public health problem worldwide. As the main vectors, mosquitoes have been classified by the Center for Disease Control and Prevention as one of the deadliest animals alive. In this ecological study, we analyzed the population dynamics of important genera and species of mosquito vectors. Mosquito immatures were collected using ovitraps and at natural breeding sites: bamboos and bromeliads. Adult mosquitoes were captured using CDC traps with CO_2_, Shannon traps, and manual suction tubes. Collections took place during the rainy and dry seasons from 2019 to 2020 in the Serra dos Órgãos National Park, Rio de Janeiro state, Brazil. The highest number of species was recorded in the ovitraps, followed by CDC and bromeliads. The breeding site with the lowest diversity was bamboo, though it showed the highest level of evenness compared to the other breeding sites. The medically important genera reported were *Haemagogus* spp., *Aedes* spp., *Culex* spp., and *Wyeomyia* spp. Culicid eggs increased in the rainy season, with a peak in November 2019 and January and February 2020, and lower abundance in the dry season, from September to October 2019. Mosquito eggs had a strong positive correlation (ρ = 0.755) with temperature and a moderate positive correlation (ρ = 0.625) with rainfall. This study shows how environmental variables can influence the ecology of disease-vector mosquitoes, which are critical in the maintenance of arbovirus circulation in a threatened biome within the most densely populated region of Brazil.

## 1. Introduction

Arbovirus infections are considered a major public health problem [1]. According to the Health Surveillance Department, Brazil recorded 761 suspected human cases of yellow fever (YF) between 2019 and 2020, as well as 603,951 probable cases of dengue, 2058 probable cases of zika, and 17,636 probable cases of chikungunya [2]. These arboviruses are transmitted to humans through bites from infected mosquitoes [3]. Mosquitoes of the genus *Aedes* spp. are important vectors of viruses that cause dengue, zika, and chikungunya, while *Haemagogus* spp. and *Sabethes* spp. are relevant YF and Mayaro virus vectors [4,5,6,7,8].

The co-circulation of dengue (DENV), zika (ZIKV), and chikungunya (CHIKV) viruses has been a burden for urban and peri-urban populations in the tropical and subtropical areas of low- and middle-income countries. A combination of poverty, rapid urban growth, and high-temperature climates sustains mosquito proliferation and the conditions for arboviral outbreaks [9,10,11]. YF is an important arboviral disease in the American continent, with most cases occurring in Brazil [12]. The territorial area of Brazil is extensive (8,510,345.540 km^2^) and has a predominantly tropical climate, with vast forested areas in the Amazon region and rainforest remnants on the eastern, southeastern, and southern coasts [13]. The Brazilian Atlantic Coast is considered a global biodiversity hotspot based on the principles of irreplaceability and vulnerability added to the extraordinary endemism of plants and high levels of habitat loss [14]. Transmission of yellow fever virus (YFV) occurs mainly in forest areas between non-human primates and infected mosquitoes; this cycle is highly endemic in the Amazon region and causes sporadic cases or outbreaks of acute febrile illness with arthropathy [8]. In terms of feeding habits and oviposition patterns, *Hg. leucocelaenus* is considered an eclectic mosquito species [15,16].

Mosquito vectors of arboviruses proliferate in a variety of breeding sites, including a wide range of available aquatic environments. In nature, transient natural breeding sites include floods, floodplains, animal burrows, coconuts, shells, and fallen leaves, whereas permanent or semipermanent breeding sites are represented by bamboo internodes and bromeliads [17]. Ovitraps are a reliable and sensitive method since they mimic these natural breeding sites; as such, they are widely used in culicid surveillance for systematic samplings conducted in the field [18]. Ovitraps are considered an excellent instrument for early detection of mosquito-borne arboviruses such as dengue, zika, and yellow fever, which are classified by the Brazilian Ministry of Health as “compulsory and immediate reportable diseases” [19].

Environmental factors appear to have a major impact on the dynamics of arbovirus transmission. Climatic factors like rainfall seem to precede ZIKV and CHIKV epidemics, high temperatures have been shown to increase mosquito population numbers, and mosquito density has been widely associated with the spread of diseases transmitted by them, such as YF [20,21]. The epidemiology of the abovementioned mosquito-borne diseases is affected by rainfall, which plays a key role in the availability of mosquito breeding sites and, hence, their reproduction and proliferation [22]. Particularly in the context of climate change, developing countries face interrelated challenges, from expanding urbanization, inadequate access to infrastructure, and mosquito-borne viruses [23]. In this study, we evaluated the association between climatic variables and mosquito vector densities, along with a comparison of different collection methods and natural breeding sites, in a federal conservation unit situated in an Atlantic Forest fragment of Rio de Janeiro state, Brazil.

## 2. Materials and Methods

### 2.1. Ethics Statement

The permanent license for the collection, capture, and transport of biological material was granted by the Biodiversity Authorization and Information System-(SISBIO)—Chico Mendes Institute for Biodiversity Conservation (ICMBio) with the number: 68206-1. All team members were vaccinated against YF.

### 2.2. Study Area

**Figure 1:**
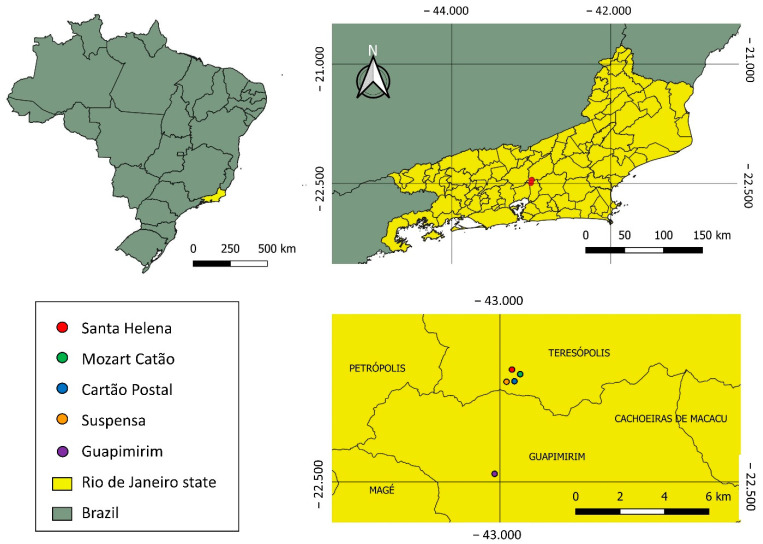
Collection sites at the Serra dos Órgãos National Park (PNSO) located in the municipalities of Teresópolis, Petrópolis, Magé, and Guapimirim, Rio de Janeiro state, Brazil.

This study was conducted in the Serra dos Órgãos National Park (PNSO), located in the municipalities of Teresópolis, Petrópolis, Magé, and Guapimirim, Brazil. The main entry point of the reserve is located near the city of Teresópolis, at Rotariana Avenue (which connects to BR 116 Rio-Bahia at km 89.5 of the municipality), with an altitude of approximately 900 m above sea level [24]. The park has an area of 20,024 hectares, and the vegetation cover is composed of a perennial hydrophilic coastal forest, also classified as dense ombrophilous forest, with a super humid temperate climate [25]. The sampling sites were as follows: Site 1 (22°26′54.0″ S 42°59′09.0″ W), Site 2 (22°27′10.8″ S 42°59′28.8″ W), Site 3 (22°27′21.2″ S 42°59′37.2″ W), Site 4 (22°27′22.3″ S 42°59′49.9″ W), and Site 5 (22°29′39.0″ S 43°00′10.0″ W) (Figure 1).

### 2.3. Mosquito Sampling

Sampling was carried out monthly from 2019 to 2020. Oviposition traps (ovitraps) were used for collecting culicid eggs. These traps consist of a black container with a capacity of 500 mL without a lid that resembles a plant pot and contains four wooden oviposition pallets (2.5 cm × 14 cm), held vertically inside the trap by a clip. Natural water and litter were added to each ovitrap in order to recreate a microecosystem similar to the natural ones. The ovitraps were distributed in five trails (Santa Helena, Cartão Postal, Mozart Catão, Suspensa, and Guapimirim trails) in the PNSO. Ten ovitraps were distributed per collection site at ground level and at 2 m; two trees at the collection sites Santa Helena and Guapimirim were chosen to install ovitraps at five heights (ground level, 3 m, 6 m, 9 m, and 12 m). Immatures were also collected from natural breeding sites: bamboos and bromeliads. Adults were collected with CDC traps with CO_2_, Shannon traps, and manual suction tubes to catch adult mosquitoes. The eggs present in the pallets of the ovitraps, along with the collected adult specimens, were sent to the Diptera Laboratory of the Oswaldo Cruz Institute in the city of Rio de Janeiro, Brazil. The positive pallets (containing eggs) from the ovitraps were separated, and the eggs were counted, following which they were immersed in transparent trays containing dechlorinated water for three days and spent three days in a dry environment. These conditions allowed us to keep the specimens alive until they reached adulthood for specific determinations, according to the methodology described by Alencar et al. (2013) [26]. The specific identification of adults was performed by direct observation of their morphological characters using a stereomicroscope and dichotomous keys following Arnell (1973), Forattini (2002), and Marcondes and Alencar (2010) [17,27,28]. The collected and analyzed specimens were listed in the Entomological Collection of the Instituto Oswaldo Cruz under the title “Coleção Mata Atlântica.” The data from the collected mosquitoes, such as the ecological indices per collection method and site, were analyzed using PAST 4.03 statistical software [29].

## 3. Results

### 3.1. Species Diversity per Breeding Site and Collection Method

A total of 6893 culicid eggs were collected, and 1975 mosquitoes were identified to the genus and species levels. The ovitrap had the highest number of species (S = 14) and the second-highest diversity index (H’ = 1.71); however, the equability index was the lowest (J’ = 0.65) compared to the other collection methods and breeding sites. There was a discrepancy in the number of individuals of each species collected, with some species being found at higher frequencies than others. The ovitraps showed a dominance of *Culex iridescens* (Lutz 1905) (44%), *Limatus durhamii* (Theobald 1901) (14.3%), *Haemagogus leucocelaenus* (Lutz 1904) (14.2%), and *Culex spinosus* (Lutz 1905) (10.5%). The second-highest richness and the highest diversity index were observed in the bromeliad (S = 11; H’ = 2.10); this breeding site also had the highest equability index (J’ = 0.88), meaning that the number of specimens from each species was similar. The most abundant species in bromeliads were *Culex intermedius* (Lane and Whitman 1951) (29%) and *Culex neglectus* (Lutz 1904) (16%). The CDC trap also showed a high richness and diversity index (S = 10; H’ = 1.74); however, this trap had a low equability index (J’ = 0.76). The CDC trap showed a particularly high abundance of *Trichoprosopon pallidiventer* (Lutz 1905) (27%). The lowest richness indices were observed in the bamboo (S = 2), Shannon (S = 4), and active capture methods (S = 7). These indices were generated considering only the mosquitoes identified at the species level (Table 1). Many medically important genera were identified in the PNSO; important vector genera found included *Culex* (50%), *Haemagogus* and *Sabethes* (11%), *Aedes* (6%), and *Wyeomyia* (6%).

### 3.2. Climatic Factors and Mosquito Abundance

**Figure 2:**
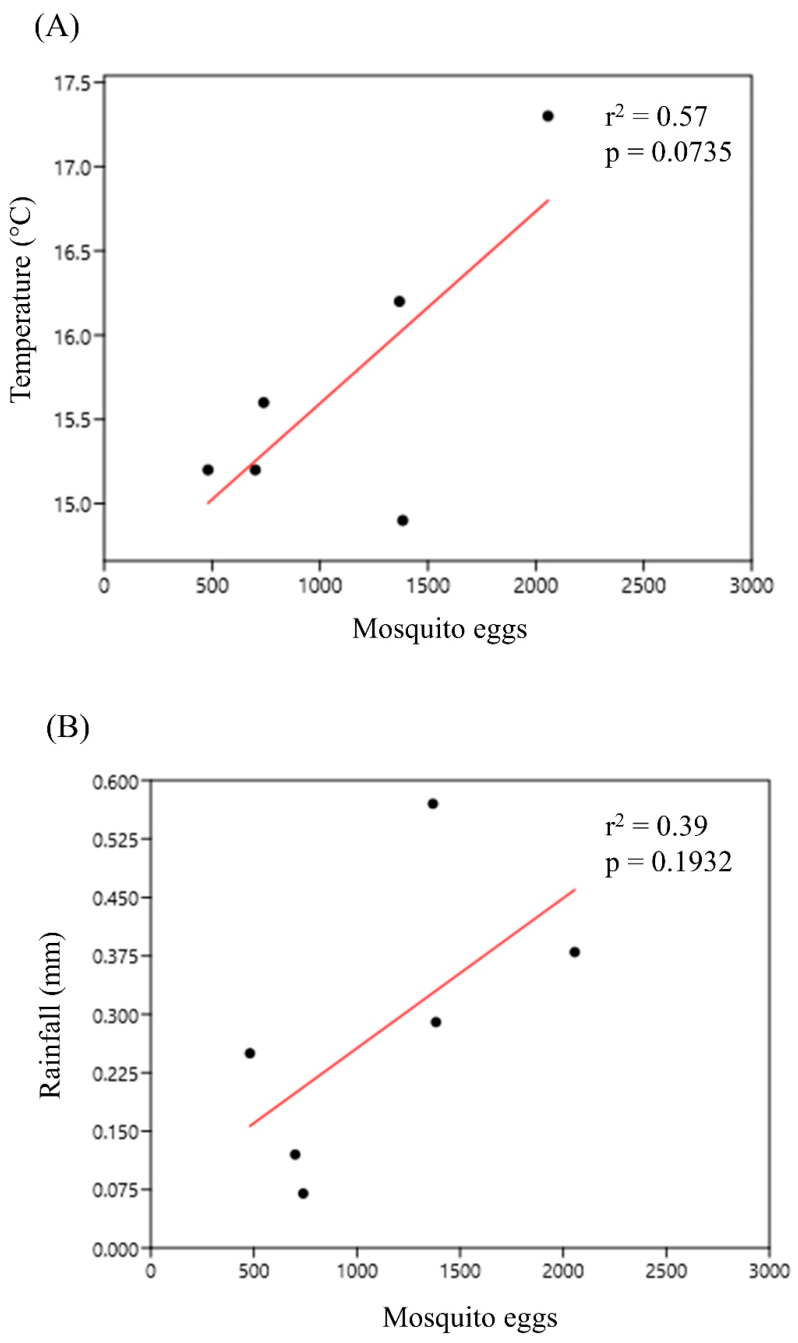
Regression analysis of the climatic factors: (**A**) temperature (°C) and (**B**) rainfall with mosquito egg abundance in the PNSO, Rio de Janeiro, Brazil.

The rainy season, which lasted from December 2019 to February 2020, had the highest number of mosquito eggs collected (4163 eggs), while the dry season, lasting from September to November 2019, had the lowest number (2564 eggs). In January 2020, there was a peak both in the total number of eggs (2056 eggs) collected and in temperature (17.3 °C). The number of eggs and rainfall peaked in November 2019, declined in December, and rose again in January 2020 similarly (INMET, 2020). Regression analysis showed that the number of eggs had a strong positive correlation with temperature (r = 0.755) (Figure 2A) and a positive correlation with rainfall (r = 0.625), although the latter was not as strong (Figure 2B). The number of culicid eggs considered for each season and associated climate factors was specific to the months of those seasons and the availability of climate data retrieved from the INMET database. The epidemiologically important vector *Hg. leucocelaenus* was more abundant during November and December 2019 and January 2020, i.e., in the summer season, when the temperatures are high and rainfalls are frequent.

### 3.3. Mosquito Abundance per Collection Site at Ground Level and in the Tree Canopy

**Figure 3:**
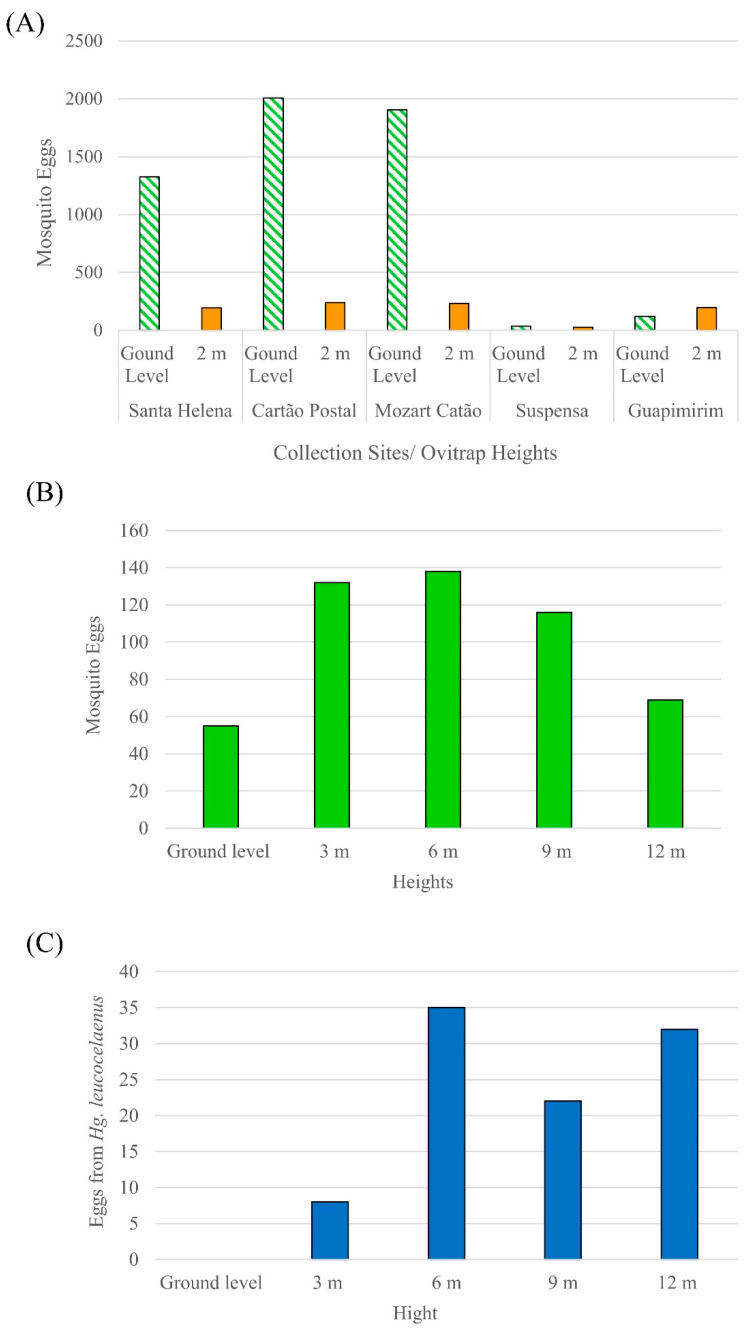
Mosquito egg abundance per collection site (**A**) and at different heights (**B**), eggs from the mosquito vector *Hg. leucocelaenus* per height (**C**).

The ovitraps at ground level had the highest number of eggs collected (*n* = 5395), or 86% of all specimens, while the remaining 14% were found in the ovitraps at 2 m (*n* = 891). Considering only the individuals identified at the species level, the collection sites with the highest number of individuals collected were Mozart Catão (*n* = 553), Santa Helena (*n* = 440), and Cartão Postal (*n* = 331). Santa Helena had the highest values of diversity index (H’ = 2.024) and equability index (J’ = 0.7475), while Suspensa had the lowest diversity index (H’ = 0.811) and a high dominance of *Cx. spinosus*. The other collection sites had similar diversity indices, with Mozart Catão presenting dominance of *Cx. iridescens* and Guapimirim of *Hg. leucocelaenus* (Figure 3A). The species that were only found in the ovitraps at ground level were *Cx. imitator*, *Cx. neglectus*, *Tx. theobaldi/pusillus*, *Tr. digitatum*, and *Tr. pallidiventer*. The species that were found at both levels but were more abundant at ground level were *Ae. rhyacophilus*, *Cx. mollis*, *Cx. spinosus*, *Cx. iridescens*, *Li. durhamii*, and *Li. pseudomethisticus*. Only two species were more abundant at 2 m: *Hg. leucocelaenus* and *Cx. pleuristriatus*. Considering the ovitraps that were distributed at five heights in relation to ground level (ground, 3 m, 6 m, 9 m, and 12 m), the highest number of eggs were observed at 3 m (*n* = 132), 6 m (*n* = 138), and 9 m (*n* = 116) (Figure 3B). The heights with the lowest egg abundance were at ground level (*n* = 55) and 12 m (*n* = 69). The medically important mosquito species *Hg. leucocelaenus* had most eggs at the heights of 6 m (*n* = 35) and 12 m (*n* = 32) (Figure 3C).

## 4. Discussion

Ovitraps have a remarkable potential for systematic sampling in longitudinal and cross-sectional ecological studies in neotropical settings [30]. In our study, this collection method had the highest number of species and the second-highest diversity index. Species of the genus *Haemagogus* spp. are known to have a clear acrodendrophilic preference [28]. Here, we show that eggs from *Hg. leucocelaenus* were more abundant in the ovitraps installed at 6 m and 12 m above ground level. Our results are quite similar to those from a study conducted on the border between the states of Minas Gerais and Rio de Janeiro, in which the authors showed that the same species had the highest frequency of eggs in traps located at the highest levels of the trees [26]. Another study performed at an Ecological Reserve (REGUA) in the municipality of Cachoeiras de Macacu showed that the species was found more frequently in ovitraps set at 5 m or higher, while species of the genus *Limatus* were usually collected at ground level [31]. This was also observed in our study, with *Li. durhamii* and *Li. pseudomethisticus* being more abundant in ovitraps set at ground level.

The highest diversity index and second-highest richness were observed in bromeliads. Several species of mosquitoes use the water accumulated in bromeliads for breeding [3]. The most abundant species in this breeding site were *Cx. intermedius* and *Cx. neglectus*; these findings were very similar to those of a study performed in the municipality of Nova Iguaçu, Rio de Janeiro, Brazil, where the dominant species were also from the same genus: *Cx. pleuristriatus* and *Cx. ocellatus*. Species of the subgenus *Microculex* are frequently found colonizing permanent natural breeding sites such as bromeliads, tree holes, and bamboo internodes [32]. In our study, *Tr. digitatum* was the only species found colonizing bamboo internodes. According to Thomas et al., bamboo internodes are one of the species’ natural breeding sites; however, they can also be found in fallen fruit husks and water storage pots [33].

The rainy season had the highest number of mosquito eggs collected, and the eggs showed a positive correlation with rainfall. These findings corroborate those of a study on the diversity of culicid vectors in an Atlantic Forest remnant also performed with ovitraps; the authors noticed that culicid diversity was greatest in the rainy season [34]. There are many studies regarding wet and dry seasons’ effect on malaria vector mosquitoes of the genus *Anopheles*; however, there are few studies on the effects of these seasons on YF vector mosquitoes of the genera *Haemagogus*, *Sabethes*, and *Wyeomyia*, like the ones described in this study [7,35,36]. We also observed mosquitoes of the genus *Aedes*, known for pathogen transmission of many arboviral diseases such as dengue, zika, and chikungunya [3,37]. Seventy-three percent of the individuals collected in the PNSO belonged to these epidemiologically important genera of mosquitoes. Since a positive and strong correlation was found between mosquito egg density with climatic variables temperature and rainfall, the wet months where the rain is heavy and temperatures are high, like December, January, and February, are expected to have high mosquito densities. The medically important vector *Hg. leucocelaenus* was reported with high densities at Guapimirim; this represents an alarming finding since this site is a touristic location of the PNSO near one of the park’s main entry points, the guardhouse, and the visitor center. The proximity of these mosquitoes to places with a high concentration of people represents a potential risk of pathogen transmission if these mosquitoes are infected and bite humans. Particular caution must be taken by visitors and workers at the park in months with higher densities of this vector. Personal protection measures against mosquitoes include the use of insect repellents, avoiding exposure to the peak of mosquito activity during the evening crepuscular period, and the use of long-sleeved shirts and pants when going into the woods. Regarding the workers who have to be exposed to these periods, the use of permethrin-treated clothing for personal protection is also recommended [38,39,40]. It is worth emphasizing that, in the present study, this species was found with higher abundance in Guapimirim during November and December 2019 and January 2020.

## 5. Conclusions

We were able to establish preliminary parameters of how environmental changes influence the ecology of important disease-vector mosquitoes, which is critical for the circulation of arboviruses in one of the most threatened biomes and most densely populated regions of Brazil. The presence of vector species in natural breeding sites and the high density of these mosquitoes in specific seasons of the year (in this case, the wet season) highlights the importance of monitoring the emergence of febrile diseases among people visiting the PNSO. A deeper understanding of mosquito ecology based on these findings and studies with a similar approach will strengthen future mosquito control strategies in Atlantic Forest ecosystems in Rio de Janeiro, Brazil.

## Figures and Tables

**Table 1:** Ecological indices of the collection methods and natural breeding sites in the PNSO from 2019 to 2020.

Mosquito Species/Breeding Sites and Collection Methods	Bamboo	Active Capture	Shannon	Ovitrap	CDC	Bromeliad
*Aedes* sp.	0	0	0	0	2	0
*Ae. (Och.) rhyacophilus*	0	0	1	91	0	0
*Ae. (Och.) scapularis*	0	19	0	0	4	0
*Culex (Cux.) mollis*	0	6	0	11	54	4
*Culex (Cux.)* sp.	0	8	13	4	117	1
*Cx. (Car.) iridescens*	0	0	0	522	5	4
*Cx. (Mcx.) aureus*	0	0	0	0	0	8
*Cx. (Mcx.) imitator*	0	0	0	4	0	4
*Cx. (Mcx.) intermedius*	0	0	0	0	0	28
*Cx. (Mcx.) neglectus*	0	0	0	2	0	16
*Cx. (Mcx.) pleuristriatus*	0	0	2	28	0	7
*Cx. (Mcx.) retrosus*	0	0	0	11	0	7
*Cx. (Melanoconion)* sp.	0	0	0	0	0	1
*Cx. (Mcx.)* sp.	0	0	0	0	0	2
*Cx. (Cux.) spinosus*	0	0	0	124	1	0
*Haemagogus (Con.) leucocelaenus*	0	0	0	167	0	0
*Limatus durhamii*	0	1	1	169	65	5
*Li. pseudomethisticus*	0	2	0	37	1	0
*Orthopodomyia albicosta*	9	0	0	0	0	0
*Sabethes (Pel.) identicus*	0	3	0	0	46	0
*Trichoprosopon digitatum*	25	6	0	1	31	0
*Tr. pallidiventer*	0	12	4	4	154	0
*Trichoprosopon* sp.	0	0	0	1	1	0
*Toxorhynchites theobaldi/pusillus*	0	0	0	2	0	0
*Wyeomyia (Pho.) edwardsi*	0	0	0	0	0	1
*Wy. (Pho.) pilicauda*	0	0	0	0	40	9
*Wyeomyia (Pho.)* sp.	0	7	4	1	54	1
Total	34	64	25	1179	575	98
Taxa (S)	2	7	4	14	10	11
Shannon (H’)	0.58	1.61	1.21	1.71	1.74	2.10
Equitability (J’)	0.83	0.83	0.88	0.65	0.76	0.88

S = Species, H’ = Shannon’s diversity index, J’ = Pielou’s equability.
